# Hospital and Institutional News

**Published:** 1913-05-24

**Authors:** 


### HOSPITAL AND INSTITUTIONAL NEWS.

### THE YORK HOSPITAL INQUIRY.

We understand that the report of Sir Cooper Perry on the inquiry recently held by him into the management of the York County Hospital will be in the hands of the governors in the course of a week. Sir Cooper Perry has gone thoroughly into the various matters, and his report runs to a very considerable length. The contents will be published after receipt by the governing body of the institution. Meanwhile, it is interesting to note that Dr. Shepherd, one of the medical officers whose charges led to the inquiry, has taken up a resident appointment at the Edinburgh Royal Infirmary. It is interesting to record that immediately after the inquiry Dr. Macqueen was offered an appointment as locum to one of the hospital staff in his private capacity.

### FROM THE PATIENTS' POINT OF VIEW.

The article published last week, "Hospitals from the Patients' Point of View," has attracted much attention. We have received a most interesting contribution from a layman, giving his experiences as patient in a London Nursing Home, and also in a Metropolitan General Hospital, which we propose to publish next week. We have also one from a Fellow of the Royal College of Surgeons, giving an account of what befell him as a patient at another Metropolitan General Hospital. We hope that these two articles may bring us many other contributions from patients with recent hospital experiences. The actual working of the Voluntary Hospital or Nursing Home, as experienced from the patients' point of view, should prove of practical value and great interest to hospital workers and managers, as well as to the public, throughout the country.

### THE OXFORD CONFERENCE.

To our preliminary notice of the coming Conference of the British Hospitals Association, which will be held at Oxford from Thursday, June 26, to Saturday, June 28, we are able to add further details of interest. Among the readers and papers arranged for will be one from Sir Thomas Oliver, M.D., Physician to the Royal Victoria Infirmary, Newcastle, and formerly medical expert to the Dangerous Trades Committee of the Home Office, on "The Treatment of Tuberculosis in the Hospitals"; and one from Mr. Keith Young, F.R.I.B.A., on "The Upkeep of Hospitals." It is hoped that Sir William Osler, Regius Professor of Medicine in the University of Oxford, will act as president of the Conference and deliver the presidential address. An opportunity will also be afforded of discussing the actual effects of the Insurance Act on the hospitals since the passing of the Act. The Rev. G. B. Cronshaw, chairman of the Radcliffe Infirmary, is most kindly taking active charge of the local arrangements, and members intending to be present will be glad to hear that a map and guide to the Oxford colleges and institutions is being prepared for their use. As usual, Thursday and Friday will be given up to papers and discussions, while Saturday will be spent in visiting the institutions or other of the innumerable places of interest. It is satisfactory to add that the Hospital Officers' Club propose to hold their dinner in Oxford during the Conference, and that the Incorporated Association of Hospital Officers are visiting Oxford on Saturday, June 28. The Association's offices are at 14 Queen Victoria Street, S.W. Mr. A. Hayes is one of the Hon. Secretaries.

### MEDICAL AID SOCIETIES FROM WITHIN.

The impasse which the Insurance Act has created in South Wales, and the crisis which has taken place at Swansea, already alluded to in these columns, makes it desirable to examine the medical aid societies which have been the instrument in bringing it about. The labour organisations bring pressure to bear on all insured persons to join a medical aid society, and import a medical man who joins the panel, and is paid a fixed salary, in addition to which house and surgery are provided. The local Insurance Committees have allowed the labour organisations collectively to make their own arrangements on these lines, and the Welsh Commissioners have declined to interfere on the ground that Section 15, Subsection 5, does not require their approval. The mode of procedure is as follows. The Insurance Committees pay the annual sum due under the Act for medical attendance on the insured to the medical aid societies, who pool it and require their medical man to attend, for 6s. 6d. per head of insured persons, not these only but their dependants; though it is true that small contributions from the latter may be thrown in. As, however, these societies have not been formed without expense, critics point out that funds may not be available to secure medical skill equal to that already provided, and, apart from this, "blackleg" labour is generally bad labour. Moreover, the medical man is placed in degrading and complete dependence on the medical aid society, to which, it may indeed be said, he sells himself when he becomes their sole and whole-time servant. More startling still, among the crop of injustices of which the Insurance Act has been the occasion, is this, that whereas liberty to make his own arrangements has been ruthlessly withheld from almost every individual applicant outside the Principality, it is being allowed collectively wholesale in Wales. Small wonder, then, that the local branches of the British Medical Association have called on the staffs of the hospitals in the affected area to withhold recognition from the men who accept such appointments. It is now announced that a deputation of medical practitioners from South Wales will be received by the Joint Commission soon after the session opens.

### A NEW HOSPITAL.

On Saturday last at Nottingham there was opened by Sir Lancelot Rolleston, K.C.B., D.S.O., a new hospital for electro-medical treatment. Its object is to devote special attention to chronic rheumatism, neuritis, and kindred obscure diseases. Small fees will be charged to those patients who are not provided with recommendations, of which subscribers of one guinea receive four, and of half a guinea two. Each recommendation holds good for a period of four weeks. It is announced that the institution will not be run for purposes of profit, but merely to cover expenses. Mr. L. Codd, a member of the committee, has presented some of the electrical apparatus, which with massage and Swedish exercises will, as the institution's name implies, play an important part in the treatment. The honorary officers are Dr. J. S. Bolton, M.D., who is medical officer; Mr. J. D. Marsden secretary; and Mr. A. E. Morton treasurer. At present the hospital will provide for out-patients only, but it is hoped to provide beds later on. It may be recalled that its foundation is the outcome of a meeting held three months ago, which Dr. Bolton convened to consider the matter.

### FRIENDLY SOCIETIES AND THE INSURANCE ACT.

The reports of the annual meetings of the great friendly societies can hardly be pleasant reading for the Government. One after another the chairmen of these societies have condemned the Insurance Act as being costly in its working and unfair to those democratic societies which have been founded and maintained by the best of the working class. The Chairman of the Manchester Unity of Oddfellows complains of "Insurance Committees who apparently cannot escape the taint of expensive officialism." The Chairman of the Hearts of Oak Society goes further and declared—apparently without a dissentient voice—that "their interests had to a great extent been sacrificed and betrayed in the interests of organisations controlled by capitalists and administered for the advantage not of the insured persons, but of shareholders and company promoters." Other societies also are complaining of the Act, although in the opinion of many outsiders the national character of it was sacrificed to the necessity of meeting with the requirements of these very friendly societies. As a matter of fact the friendly society members were doing for themselves voluntarily what the Insurance Act wished to do compulsorily, and a good deal more. It is hard to see any good reason why they should have been interfered with in their career of well-doing. The State had a case for compelling the thriftless and improvident to make provision for their time of need, but not for laying down rules for those who had already shown thrift and foresight. But the truth is that the lives of the friendly society members —healthy, prudent, and industrious—were needed to strengthen the funds and enable the promoters of the Act to make a brave display of promises. The doctors spoke the truth when they explained that to take the whole of the insurable population at the rate at which they took the members of friendly societies was impossible, and they were maligned and mocked at in consequence. The Act is not yet a year old in its working and already those who pushed it through have to promise an amending Act. Let us hope that when that Act is brought forward its promoters will listen to honest criticism, and not assume that everyone who sees a flaw in it is sordid and selfish and hates the poor. But at present the Act seems to satisfy no one, except perhaps the army of officials who have found places under its sheltering wing.

### HOSPITALS AND THE BRITISH MEDICAL ASSOCIATION.

A month after the Conference of the British Hospitals Association at Oxford, that is to say, on July 22, the annual meeting of the British Medical Association will take place at Brighton. This year more than usual interest for institutional workers will attach to the proceedings, since papers on "Hospitals in Relation to the Stage, the Public, and the Medical Profession" will be read by Sir Henry Burdett and Dr. J. G. Gibbon. Among others the problems involved in providing hospital accommodation for insured persons under the Insurance Act will be discussed. The public generally will be interested in the discussions of the Section of Climatology and Balneology, when the international aspects of British health resorts and sea-bathing will be considered. The eternally debated aspects of crime and punishment will be among the papers read in the Section of Medical Sociology, when Dr. Charles Mercier, Sir B. Donkin, and Dr. James Scott will be among the readers. Sir James Barr is the president, and the president-elect is Dr. W. A. Hollis, consulting physician to the Sussex County Hospital, Brighton.

### RANDOM MEMORIES OF SIR E. HAY CURRIE.

In our last issue, referring to the death of Sir Edmund Hay Currie, the secretary of the Metropolitan Hospital Sunday Fund, we mentioned that he was fond of relating stories of his experiences in the Crimea. It was in 1854 that he went to the Crimea, as a friend assures us, in connection with the officers' commissariat, and arrived there just as the wounded were being landed from Inkerman. His brother was badly shot at the time. Sir Edmund used to recall how he and John Macdonell, who was printer to the *Times* in the Parnell trial, used to cross the Bosphorous in a caique every morning for four months. He was for a similar period at Constantinople, and used to enlarge on the mismanagement of the 10,000 sick and wounded at Scutari. Another of his memories was the smallpox epidemic of 1870, when all the hospitals were full. Sir Charles Dilke was President of the Local Government Board at the time, and consulted with Sir Edmund as to how it would be possible to empty the hospitals and to make London habitable again. Sir Edmund, as chairman of the General Purposes Committee of the Metropolitan Asylums Board, replied that sick camps must be established, and eventually himself took large numbers of persons to Dartford, where camps were formed for 500 men and women apiece. The transition was carried out in horse vans by night, and the necessary change was made at Bexley Woods. After this had been accomplished Sir

Edmund returned home, when his servant contracted smallpox; he was immediately fixed on as the source of infection, which, however, further investigation proved to be the Sunday class which the servant attended. The smallpox patients, who were of the class of public-house corner men, were very troublesome. There were at the time three ships in the Thames, including the *Atlas* and *Endymion*, and when the former was sent to fetch Dilke, the surgeon on board at first refused to admit him on the ground that he had not been vaccinated. These random memories, which are based on a few notes taken after a conversation some time ago, are printed without emendation. For though errors of detail may have crept in, the interest is rather a personal than a historic one, and gives a glimpse of a personality which is well worth a larger memorial. Sir Edmund's Life was once, we believe, undertaken by a lady, but if our information is correct, the project never came to fruition.

### A LADY SECRETARY OF A GENERAL HOSPITAL.

The new St. Andrew's Hospital, at Dollis Hill, to which we alluded last November when the building was in course of construction, has now been opened for the reception of patients. It may be recalled that the hospital is the outcome of a gift of £100,000 placed in the hands of the Cardinal Archbishop of Westminster by a lady who desired to remain anonymous during her lifetime. It says little for those responsible that this vast sum has not proved sufficient to meet the cost of erecting and equipping the hospital, and it commences its career to some extent in debt. Further, this £100,000 will not provide one free hospital bed for the sick poor, and it will receive only paying patients for the present. The fees are 2½ guineas a week for ward patients (there being nine beds in some wards, eighteen in others), and 7 guineas a week for patients having private rooms. These charges include all attendance by the resident medical officer and the visiting staff, as well as the fee for operation, if such should be necessary. The secretary resides on the premises, and the institution is probably unique amongst general hospitals in having a lady to fill this post.

### AN UNPLEASANT EPISODE AT WANDSWORTH.

The Wandsworth Board of Guardians has decided that in future all officers appointed by them shall be subject to a satisfactory medical report from the superintendent medical officers. The resolution is due to a somewhat unpleasant episode that occurred in the appointment of an officer. The testimonials and qualifications were highly satisfactory, but a telephonic communication was received from the matron of an institution that the applicant for the position was suffering from consumption, and, consequently, acting on this report, the Guardians gave the post to another. An endeavour was made to induce the board not in future to ask for a private report, and not to act upon it if one was sent in, but the Guardians declined to accept the proposal, agreeing to abide by the reports of their own medical staff.

### ADMINISTRATIVE REFORMS AT WANDSWORTH INFIRMARIES.

In view of the recent articles and correspondence in The Hospital suggesting schemes of revised administration it is interesting to note that the Wandsworth Board of Guardians have adopted a scheme for the reorganisation of the medical staff at their infirmaries. In 1910 the Local Government Board, when sanctioning the appointment of the medical staff at the new St. James' Infirmary, pointed out that when practicable one medical officer should be in principal charge of the medical work at the institutions under the Guardians' control. The Board are enabled through the resignation of Dr. J. B. Neal, medical superintendent of St. John's Hill Infirmary, to fall in with this suggestion. They propose, with the sanction of the Local Government Board, that Dr. W. L. Maccormac, at present superintendent of St. James' Infirmary, should act in the same capacity at St. John's Hill Infirmary, his salary for both institutions being £600 a year, rising to £700 by annual increments of £25. Dr. Essex F. W. Nixey is to be deputy medical superintendent at St. John's Hill Infirmary at a salary of £300 a year, to be increased by annual increments of £20 to £400. Other changes include the increase in the salary of Dr. Keats, senior assistant medical officer at St. James' Infirmary, from £200 to £250, and the appointment of a senior assistant medical officer at St. John's Hill Infirmary at a commencing salary of £170 per annum, increasing to £200.

### MANSION HOUSE DINNER FOR THE LONDON HOSPITAL.

It is announced that the Lord Mayor, Sir David Burnett, will preside at a dinner in the Mansion House some time during June in aid of the London Hospital Quinquennial Appeal. The special feature of the Lord Mayor's letter asking for support is its admirable succinctness. Not a word is wasted, and yet everything that matters is there. Successive Lord Mayors have vied with their predecessors in promoting, each, some super-excellent scheme of mercy; and we do not think that the Lord Mayor need fear that the chosen object of his endeavour will fall short of any selected in recent years.

### HONOUR FOR CARDIFF HOSPITAL MANAGERS.

We are glad to note that Colonel Bruce-Vaughan, the chairman of the board of management of King Edward VII.'s Hospital, Cardiff, has been made a Justice of the Peace. In addition, two other members of the board, Mr. Sam Fisher and Mr. Leo Joseph, have received the same distinction. This undoubtedly has been deserved by all three, and in the case of one at any rate the distinction has been long overdue. At the same meeting, at which the congratulations of the board were given to the three new magistrates, the question was raised as to whether a man found with an in-patient ticket upon him, who had been fined for a breach of the law, was a fit person for treatment at the hospital. Mr. L. D. Rea, the secretary, at once pointed out that accident cases were admitted at all hours without question, and he was thankful to think that the character of each person was never considered. A hospital exists to treat physical and not moral ills, and surely none who are lamenting the problems arising out of the inquiries and classification necessitated by recent legislation would wish to add to a secretary's troubles the additional onus of moral judgment. That indeed would be the final end of hospital treatment, and is unthinkable under the voluntary system. "Without distinction of creed" has long been our boast. Without distinction of character is its corollary and implication.

### NATIONAL SANATORIUM ASSOCIATION.

PRESIDING at the seventh annual meeting of the National Sanatorium Association, held on Saturday last, at Delegate Hall, Hearts of Oak Buildings, N.W., Mr. T. E. Durrant, chairman of the Council, said that ten additional beds had been taken up last year, and the number at present endowed, on a yearly basis, was ninety-four. Since the National Insurance Act had become operative on July 15 last, the Council had received applications from nearly every Insurance Committee in England for beds; in fact, it was quite safe to say that, if they had the accommodation, considerably over four figures could have been allocated to Insurance Committees who desired the accommodation of the National Association. He had heard that a sum of one and a half million pounds had been set aside for the purpose of further sanatoria work, and he hoped that the work done by the National Association would not be overlooked by the Government when they were considering how those grants should be awarded. Seventeen beds, he thought, were at present available for Insurance cases. Necessarily, he added, the Council had had to charge a slightly increased rate for these beds, inasmuch as no capital expenditure had been contributed toward the beds of the Insurance Committees. If, however, they received a grant from Government toward the capital expenditure, then he thought he might say that the question of receiving National Insurance patients at the same rate as that of friendly societies would be entertained by the Council. Of the 300 patients discharged, 181 had their disease arrested; 91 "improved"; and 23 "unimproved"; while five deaths only occurred.

### THE TUBERCULIN TEST.

A BLUE-BOOK, written by Dr. L. Cobbett and Dr. A. Stanley Griffith, was issued on Monday as a supplement to the appendix to the final report of the Royal Commission appointed to inquire into the relations of human and animal tuberculosis. A summary of recorded facts in connection with tuberculin testing of lower animals supports the claim that the tuberculin test was constant enough to form a practical means of diagnosis so far as the large domestic animals are concerned. It proved, however, very inconstant in the case of tuberculous animals of the other species investigated. The farm investigator as well as the clinical physician should find the volume full of suggestion.

### NEW MEDICAL OFFICER OF THE CHARTERHOUSE.

THE Governors of Sutton's Hospital in Charterhouse, London, have appointed Mr. Guy Wood, M.B., M.R.C.S., L.R.C.P., to be resident medical officer in place of the late Mr. A. Chune Fletcher. Sutton's Hospital, it will be remembered, provides a home and maintenance "for gentlemen reduced by misfortune." Some fifty pensioners are supported. Mr. Wood, who is a graduate of Durham University and took the Conjoint Diploma in 1890, studied at University College, London. He formerly held the post of senior assistant medical officer at Rainhill County Mental Hospital.

### THE SALE OF EAST HASTINGS HOSPITAL.

AFTER a long discussion the Hastings Town Council has decided to purchase the East Sussex Hospital, on the motion of the Deputy-Mayor, Mr. Clement Hill, for £15,000. This was eventually carried by thirty votes to three, after an amendment that the governors of the hospital be asked to offer the site at a reasonable price had been moved and withdrawn. At this point it is interesting to consider the progress which the King Edward VII. Memorial Fund has made, the object of which is to rebuild the East Sussex Hospital on a more suitable site. At the present time, including the purchase money of the original hospital, four-fifths of the £50,000 estimated as necessary to complete the memorial have been subscribed, and the actual building will be shortly undertaken. It is hoped, indeed, that the £10,000 needed will be subscribed before the completion of the new institution so that the hospital's new lease of life may start free from debt. The public, with this opportunity before them, should remember one pregnant fact. The policy of rebuilding in its entirety a new hospital is a policy of courage, but in almost every case has proved one of economy. We could have no difficulty in proving this by showing in several glaring instances how enough money has been spent on tinkering with obsolete buildings over a period of eight years or so to rebuild the institution almost twice over. The public attached to and interested in the practical bearings of economy with efficiency in connection with the East Sussex Hospital should bear this in mind, for in no department of architecture has obsolescence been more quick than in hospital design and construction.

### LONDON'S LATEST MATERNITY OUT-PATIENT DEPARTMENT.

WHAT will appear to the public to be the first practical step in the establishment of the new out-patient department at the Royal Free Hospital, Gray's Inn Road, took place on Monday when Princess Christian laid the foundation-stone of the Helena Building, which is the name of the additional wing. Mr. H. V. Ashley prepared the sketch-plans, which provide for a comprehensive grouping of departments, including a casualty unit on the ground floor, a maternity department, and special departments, with medical students' and wardmaids' quarters above. Lord Sandwich, the chairman of the committee, alluded to the widening sphere of usefulness opening out for medical women and to the consequent need for extending the only hospital in London through which no men can enter the medical profession.

### THE JUBILEE OF THE HOME FOR INCURABLES.

The Secretary of the British Home for Incurables, Mr. Edgar Penman, announced at the annual meeting last week that £20,000 has been subscribed towards the Jubilee Commemoration Fund. It is now fifty years since Queen Alexandra granted her patronage to the institution, which was the first in England to receive this honour. In order, however, to complete the fund a further £10,000 is required, and early in July it is hoped that Queen Alexandra will open the new wing, named after her, which forms part of the Jubilee celebration scheme. Lord Strathcona, the president of the institution, who presided, received a very warm tribute from Mr. F. A. Bevan, the chairman of the board of management, who emphasised his practical interest in the work, and said he was "not a mere figure-head president." Where such a compliment is deserved, as in this case, it is one which should be appreciated as much by the supporters of a hospital as by the president, and we hope that the latter will prove worthy of his example by completing the Jubilee Fund before the date of Queen Alexandra's visit.

### POISONED CONFECTIONERY IN HOSPITALS.

In the third of his series of lectures on drugs at the City of London School, Dr. F. M. Sandwith, Gresham Professor of Physic, gave some interesting details of the historical uses to which arsenic has been put as a poison. The famous Marquise de Brinvilliers, the extensive poisoner of Louis XIV.'s time, he reminded his listeners, studied the toxic effects of arsenic by giving poisoned confectionery to the poor whom she visited in the hospitals of Paris. Finding this successful, she repeated her experiments, also with success, on her father and two brothers. In view of a recent murder trial it is curious to note Dr. Sandwith's statement that twenty-four cases of poison by means of fly-papers are recorded, seven of which were described as homicidal. The lecturer added that homicidal poisoning by arsenic was declining to a large extent owing to the restrictions on the sale of poisons. In contradistinction to this side of the question the beneficial effects of three modern arsenical preparations were alluded to—atoxyl, soamin, and salvarsan. Sinister laymen have occasionally accused the profession of experimenting on poor patients in hospitals in the past, but of nothing so appalling as the doings of the marvellous Marquise: what a new light they throw on the opportunities of a hospital visitor!

### CAN INSURANCE FEES BE GARNISHEED?

A remarkable case was heard at Lambeth County Court on Tuesday, when the question was raised as to whether the fees paid to a doctor under the Insurance Act could be garnisheed. It appeared that the London Insurance Committee had paid into Court £33 due in fees to Dr. Michael, of Camberwell Road, S.E., under a garnishee order, and an application was made to Judge Parry for the amount to be paid out to meet the costs in a High Court action, in which Dr. Michael was involved. Judge Parry said that it was a mortal thing for a doctor if he had the power to make an order for the sum to be paid out of Court, especially for a doctor taking cash in small fees. Of course, he added, in the old days it was practically impossible to garnishee such a man's income. If he had to make the order when a doctor was on the panel all his creditors had to do was to garnishee his fees, and the doctor could either starve or go to into the workhouse. It seemed rather a hard case, and he preferred to consider the matter further. Even if he had no choice but to make the order, the question arose whether he had to make the order for the whole amount of the money or for half or two-thirds to be paid.

### PATIENTS AND SMOKING.

At the Cameron Hospital, West Hartlepool, Durham, the recent regulations prohibiting patients from smoking was sharply criticised by certain members of the Board. Mr. W. Stubbs stated that smoking had been allowed in the hospital for many years and that "when a man was accustomed to smoking it was as important to him as his food." Mr. J. G. Taylor, the secretary, reminded the critics that the matter had been referred to the medical staff four months ago, and that they were unanimously in favour of its prohibition. Dr. Gamlen, apparently to the surprise of some of his hearers, remarked that it was the exception and not the rule to allow smoking in hospitals, and Mr. Beckett asked if there were any room away from the ward were smoking might be permitted. The Chairman, Mr. W. Brankston, said there was no room for the purpose, which provides another illustration of the passing of the day-room, referred to last week, owing to the shortened stay of patients in the wards. This being the fact, and convalescents being sent home or away at the earliest moment, it is surprising that the question of smoking in the wards should have been resurrected in this fashion.

### TWO LARGE GIFTS TO PROVINCIAL HOSPITALS.

We have to record this week two gifts of exceptional importance. to provincial hospitals. The first is the bequest of the late Mr. William McEwan, formerly member of Parliament for Central Edinburgh, of £15,000 to the Edinburgh Royal Infirmary, with an additional £1,000 to the convalescent home connected with the institution at Corstorphine. The second is Lord Tredegar's presentation to the Newport and Monmouthshire Hospital of the Friars mansion and its ground, which extend to fourteen acres, and immediately adjoin the hospital site. Of the Friars Estate the site of the hospital once formed part, and the uncle of the late Viscount, Mr. Octavius Morgan, was formerly occupier. The Friars will probably be used as a convalescent home.

### AN EXPERIMENT IN HOSPITAL VISITATION.

Certain Nonconformist contemporaries have drawn attention to the success which has attended a recent experiment in Manchester for the organised visitation of Wesleyan patients at the Royal Infirmary, and as the problems for each denomination in this matter are largely similar we give the main points of the experiment. The work was begun haphazard when the Royal Infirmary was opened four years ago by a minister of a neighbouring chapel and his successor. The need then became more fully understood, and ministers of three neighbouring circuits agreed to give a week in turn. This made, however, the extent of the work to be done wholly apparent, and a visiting chaplain, who is paid £80 a year, plus some £20 for expenses and postage, was appointed by the synod, under a committee of management, which included medical men, a secretary and treasurer, and the visiting chaplain himself, the Rev. R. Simpson. The arrangement was announced on all the circuit plans in the district, and notifications of patients desiring visits were invited. After six months' work the six institutions covered by the plan have provided 891 Wesleyan patients. The aim has been to visit each patient at least once a week, and thus the number of visits paid is much higher than the number of patients. The visiting chaplain keeps a register of each case, and often informs the minister of the return home of the patient. The plan has not been found to clash with the visiting of patients by their own ministers as occasion offers. The salary and expenses are provided for by private subscriptions and grants from the quarterly meetings. The committee of management meets occasionally, and the sub-committee receives the chaplain's reports once a month. The plan offers scope for almost infinite extension, and may lead other denominations to imitate what appears to be a generally satisfactory attempt to make this side of hospital work as completely organised as are its others.

### POOR-LAW DISPENSER'S EXCEPTIONAL SERVICES.

"Nothing succeeds like success," and the Southwark Board of Guardians have reaped the truth of the old proverb due to their persistency. They desired to increase the salary of their dispenser, Mr. J. F. Dunstan, from £180 to £190 per annum, but the Local Government Board pointed out that it was only a little over two years ago since Mr. Dunstan reached his maximum salary, and desired to know what exceptional circumstances might justify a further increase. The reply of the Guardians was that Mr. Dunstan had been in their service since 1893, with the exception of a brief interval, and that his duties included dispensing for the outdoor poor, for four medical officers and two workhouses with 955 inmates. The latter facts were sufficient to induce the Local Government Board to agree to the increased salary.

### NEW HOSPITAL MATRONS: TWO IMPORTANT APPOINTMENTS.

The vacancy in the matronship at St. Thomas's Hospital caused by Miss Hamilton's retirement on a pension from ill-health will, it is stated, be offered to Miss Alicia Lloyd Still, who has done such invaluable service in the cause of modern hospital and nursing administration, first at the Brompton Consumption Hospital and latterly in her present post of lady superintendent at the Middlesex Hospital. No better selection could have been made by the Governors of St. Thomas's Hospital, for Miss Lloyd Still is full of knowledge and consideration for her staff. She is, too, deservedly popular with the medical profession. The present assistant matron at St. Thomas's Hospital, Miss F. Redn, has, we hear, been appointed matron to the Brompton Hospital for Consumption. Miss Redn has rendered devoted service to St. Thomas's Hospital, where she was trained, and her work there as assistant matron has been very greatly appreciated by the treasurer, the medical and nursing staff.

### THIS WEEK'S DRUG MARKET.

The Drug market is slow in recovering from the effects of the recent holidays and business continues very quiet. The most interesting item of news relates to the progress of the negotiations which have for a long time been going on between the planters of cinchona bark in Java and the European makers of quinine. It is now stated that an agreement has been arrived at, the effect of which will be to steady the price of bark and consequently of quinine. Full confirmation of this statement is expected shortly. As a result of this arrangement it is expected that the price of quinine will be advanced, but to what extent cannot be estimated at present. Cod-liver oil is an exceedingly quiet market, but the price is practically unchanged in the absence of business. The results of the fishing have been better during the past few weeks. There is no alteration in the position of opium. Menthol is tending lower in price. Orange oil and essence of lemon are dearer. The value of ergot of rye has a lower tendency. Saffron is advancing in price. There is no change in the position of cocaine, but it is still thought probable that the price may be advanced.

### TO OUR READERS.

Contributions are specially invited from any of our readers to these columns. They should deal with topical subjects and news. They must be authenticated for the information of the Editor only. The minimum payment if published is 5s. There is no hard-and-fast rule as to space, but notes of about twenty lines in length are preferred.

